# Supplementary material for: Perception of urban subdivisions in pedestrian movement simulation
Source: PLoS One. 2020 Dec 31;15(12):e0244099. doi: 10.1371/journal.pone.0244099 (PMC7774988; doi:10.1371/journal.pone.0244099)
Supplement: S1 Appendix — (PDF) [file pone.0244099.s001.pdf]

S1 Appendix - Supplementary figure

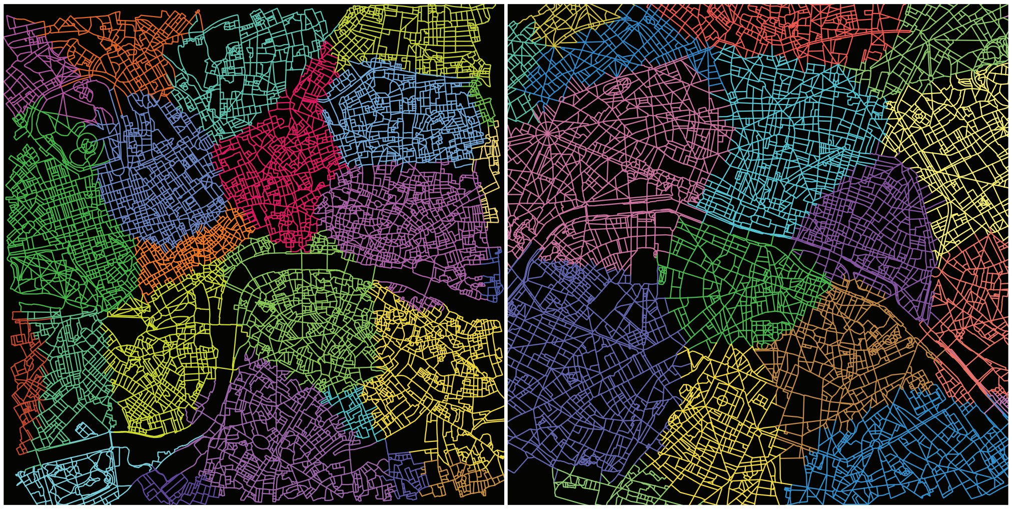

S1 Fig. Regions extracted from the street network of London (United Kingdom), left, and Paris (France), right, employing the modularity optimisation algorithm. Different colours indicate different regions. Data source (street network): OpenStreetMap data [1].

Reference

1. OpenStreetMap contributors. Planet dump retrieved from https://planet.osm.org; 2020. Available from: `https://www.openstreetmap.org`.
